# Unravelling the role of gut microbiota in acute pancreatitis: integrating Mendelian randomization with a nested case–control study

**DOI:** 10.3389/fmicb.2024.1401056

**Published:** 2024-07-03

**Authors:** Chang Qu, Jiongdi Lu, Yongyan Chen, Jia Li, Xiaoqing Xu, Fei Li

**Affiliations:** ^1^Department of General Surgery, Peking University First Hospital, Peking University, Beijing, China; ^2^Department of General Surgery, Xuanwu Hospital, Capital Medical University, Beijing, China; ^3^Department of Epidemiology and Biostatistics, School of Public Health, Peking University Health Science Centre, Beijing, China

**Keywords:** gut microbiota, acute pancreatitis, Mendelian randomization, nested case–control study, *Bacteroides plebeius*

## Abstract

**Background:**

Gut microbiota may influence the development of acute pancreatitis (AP), a serious gastrointestinal disease with high morbidity and mortality. This study aimed to identify a causal link by investigating the relationship between gut microbiota and AP.

**Methods:**

Mendelian randomization (MR) and a nested case–control study were used to explore associations between gut microbiota composition and AP. 16S rRNA sequencing, random forest modelling (RF), support vector machine (SVM), and Kaplan–Meier survival analysis was applied to identify significant gut microbiota and their correlation with hospitalization duration in AP patients.

**Results:**

Bidirectional MR results confirmed a causal link between specific gut microbiota and AP (15 and 8 microbial taxa identified via forward and reverse MR, respectively). The 16S rRNA sequencing analysis demonstrated a pronounced difference in gut microbiota composition between cases and controls. Notably, after a comprehensive evaluation of the results of RF and SVM, *Bacteroides plebeius* (*B. plebeius*) was found to play a significant role in influencing the hospital status. Using a receiver operating characteristic (ROC) curve, the predictive power (0.757) of *B. plebeius*. Kaplan–Meier survival analysis offered further insight that patients with an elevated abundance of *B. plebeius* experienced prolonged hospital stays.

**Conclusion:**

Combining MR with nested case–control studies provided a detailed characterization of interactions between gut microbiota and AP. *B. plebeius* was identified as a significant contributor, suggesting its role as both a precursor and consequence of AP dynamics. The findings highlight the multifactorial nature of AP and its complex relationship with the gut microbiota. This study lays the groundwork for future therapeutic interventions targeting microbial dynamics in AP treatment.

## Background

The human gastrointestinal tract harbors a diverse and complex microbial community known as the gut microbiota. The human gut microbiota, consisting of approximately 10 to 100 trillion microorganisms, is estimated to outnumber human cells by ten-fold (Bäckhed et al., 2005). This implies that as a collective “superorganism,” our bodies are composed of 10% human cells and 90% microbial cells (Zhao, 2013). Furthermore, the collective genome of gut microbiota, known as the gut microbiome, contains approximately 150 times more genes than the human genome (Qin et al., 2010). Previous research has elucidated numerous functional characteristics of the gut microbiota, including fermentation of indigestible dietary polysaccharides, the synthesis of essential amino acids and vitamins, and the metabolism of xenobiotics (Yatsunenko et al., 2012; Cabreiro et al., 2013). In this study, ‘gut microbiota’ refers to the community of microorganisms in the digestive tract, while ‘gut microbiota composition’ describes the specific types and proportions of these microorganisms. Factors such as genetics, nutrition, lifestyle, disease, and age can influence the composition of the gut microbiota. Recent studies have further highlighted the role of gut microbiota in modulating immune responses and metabolic processes, this community has come under scientific spotlight due to its wide-ranging impact on host health (Li et al., 2016). Acting as an essential metabolic and immune hub, the gut microbiota orchestrates pivotal processes, from digestion and nutrient absorption to modulation of immune responses (Zmora et al., 2019). Far from being merely bystanders, these microbes have been implicated in many diseases and serve as modulators of many pathophysiological pathways.

AP is the most common gastrointestinal disorder and requires urgent hospitalization. AP has an annual incidence of 34 cases per million people in high-income countries (Xiao et al., 2016). Approximately 20% of patients develop moderate to severe AP, accompanied by pancreatic or peripancreatic tissue or organ failure, or both, with mortality rates ranging from 20 to 40% (van Sandwort et al., 2011; Nicolien et al., 2019)^.^ AP is a serious gastrointestinal challenge caused by rapid pancreatic inflammation (Iyer et al., 2020). With the considerable morbidity and mortality associated with AP, there is an urgent need to examine the diverse factors influencing its pathogenesis, particularly in light of the growing evidence highlighting the instrumental role of the gut microbiota in its genesis and progression (Wang et al., 2022).

Although contemporary research has drawn connections between the gut microbiota and AP, a definitive causal relationship remains elusive (Wang et al., 2023). This poses a fundamental conundrum: Are the perturbations in the gut microbiota implicated in the development of AP, or does AP itself induce shifts within the microbial community? This dynamic interplay between the two is gaining traction in scientific discourse, with emerging opinions suggesting that microbial shifts might not merely be consequential but could also act as potential instigators of AP.

MR analysis leverages common genetic variations related to modifiable environmental exposures to investigate potential causal relationships between these exposures and diseases (Sanderson et al., 2022). This approach is widely used because it mitigates issues inherent in observational studies, such as confounding factors and reverse causation, by using genetic variants as instrumental variables (IVs). Two-sample MR analysis is particularly effective, as it combines SNP-exposure and SNP-outcome associations from separate genome-wide association studies (GWASs) to produce a single causal estimate. By leveraging genetic variants as IVs, this method promises a clearer perspective, potentially bypassing confounders that may have clouded previous evaluations. The increasing availability of large-scale summary statistics from GWASs on gut microbiota and various diseases has significantly enhanced the statistical power of this method. By employing MR, this study aims to provide robust evidence of causality, clarifying whether shifts in gut microbiota composition are a cause or effect of AP. This method enables the precise identification of microbial taxa that influence AP pathogenesis, thereby offering a solid basis for understanding the disease’s mechanisms and identifying potential therapeutic targets.

The intricate relationship between the gut microbiota and AP has been a focal point of debate in the global scientific community. Although there is a prevailing consensus suggesting decreased microbial diversity in patients with AP, discussions continue on fluctuations in specific microbial taxa and their clinical significance. In response to this nuanced landscape, our study employed a comprehensive, multi-faceted approach that integrates MR with a nested case–control study design. Furthermore, we screened the key microbial taxa in patients with AP and performed a prognostic analysis of their relationship with the duration of hospitalization. Taken together, our research aimed to elucidate the intricate relationship between the gut microbiota and AP and offers a revitalized perspective, while delineating a rationale for future clinical investigations.

## Methods

### Study design and sample collection

In this study, we used a combined approach of MR analysis and nested case–control research to investigate the relationship between the gut microbiota and AP. We retrospectively analyzed the clinical data of patients with AP admitted to the Department of General Surgery of the Xuanwu Hospital, Capital Medical University between January 2014 and December 2020. The inclusion criteria for patients with AP included the following: computed tomography/magnetic resonance imaging and clear evidence for the presence of pancreatic and/or peripancreatic necrosis. Patients with (i) oedematous pancreatitis, (ii) acute exacerbation of chronic pancreatitis, or (iii) emergency surgery owing to complications associated with AP were excluded. Stool samples were collected from patients with AP after treatment. Sixty-eight patients with AP and 20 healthy controls were included in this study.

### Data sources for Mendelian randomization

The MiBioGen consortium assembled a cohort of 18,340 individuals from various ethnic groups, such as European, Latin/American, Hispanic, and East Asian (Kurilshikov et al., 2021). This collection provides unparalleled insight into the genetic influences on the human gut microbiota. For the microbiome quantitative trait loci (mbQTL) mapping analysis, only the taxa found in a minimum of 10% of the samples were included, amounting to 211 taxa across nine phyla, 16 classes, 20 orders, 35 families, and 131 genera. Full details of the association study are available at www.mibiogen.org. In this study, 15 unnamed bacterial traits were excluded, resulting in a refined set of 196 bacterial traits for subsequent analysis. Genome-wide association studies (GWAS) summary statistics for AP were obtained from the FinnGen consortium, specifically from the R9 release. This dataset includes 3,022 cases and 195,144 controls for AP and is available at https://risteys.finregistry.fi/endpoints/K11_ACUTPANC. All selected GWAS for AP from the FinnGen consortium were approved by the FinnGen Steering Committee, and informed consent was obtained from the individuals involved. The R codes and analysis files have been uploaded to a GitHub repository.^1^

### Instrumental variable selection

IVs were filtered using a threshold of *p* < 1 × 10^−5^ to produce a more comprehensive result. Subsequently, these IVs were subjected to linkage disequilibrium (LD) with parameters set at r^2^ = 0.01 and a distance of 10,000 kb, a process aimed at minimizing correlations among single nucleotide polymorphisms (SNPs). Any SNPs that demonstrated inconsistent alleles between the exposure and outcome samples, as well as palindromic SNPs with intermediate allele frequencies, were excluded from the analysis. The robustness of the remaining SNPs was assessed using F-statistics.

### DNA extraction and 16S rRNA gene sequencing

Each stool sample was snap-frozen in liquid nitrogen following collection and was stored in a sterile container in a − 80°C freezer until use. The basic procedure for 16S rRNA sequencing involved extracting DNA from experimental samples, amplifying a specific variable region of the 16S rDNA, and sequencing it using high-throughput sequencing.

Fresh faecal samples of patients hospitalized within 1 week and fresh faecal samples from patients with AP who met the inclusion criteria during outpatient follow-up were also retained. The samples were stored in a − 80°C freezer (faecal exposure time: 2 h). Microbial DNA was extracted using the E.Z.N.A.^®^ Soil DNA Kit (Omega Bio-Tek, Norcross, GA, United States) following the manufacturer’s instructions. The final DNA concentration and purity were determined using a Nanodrop 2000 UV–visible spectrophotometer (Thermo Scientific, Wilmington, MA, United States). DNA quality was assessed using 1% agarose gel electrophoresis.

Genomic DNA was extracted from the samples using the Cetyltrimethylammonium Bromide (CTAB) method. DNA concentration and purity were assessed using 1% agarose gel electrophoresis. The DNA was diluted to a concentration of 1 ng/μL using sterile water. The 16S rRNA gene regions (16S V3–V4) were amplified using specific primers 341F (5′-CCTAYGGGRBGCASCAG-3′) and 806R (5′-GGACTACNNGGGTATCTAAT-3′). All PCR reactions were performed using 15 μL of Phusion^®^ High-Fidelity PCR Master Mix (New England Biolabs), with forward and reverse primers at a concentration of 2 μM each and approximately 10 ng of template DNA. The thermal cycling conditions were as follows: initial denaturation at 98°C for 1 min, denaturation at 98°C for 10 s, annealing at 50°C for 30 s, extension at 72°C for 30 s. The final extension step was performed at 72°C for 5 min.

Equal volumes of PCR products were mixed with 1X TAE buffer and subjected to 2% agarose gel electrophoresis. The PCR products were mixed in equimolar ratios and purified using a universal DNA purification kit (Tiangen, China).

Sequencing libraries were generated using the NEB Next^®^ Ultra DNA Library Prep Kit (Illumina, United States) according to the manufacturer’s recommendations, with index codes added. The quality of the library was evaluated using an Agilent 5,400 Bioanalyzer (Agilent Technologies). Finally, the libraries were subjected to Illumina NovaSeq sequencing.

The DADA2 plugin in the Qiime2 software was used to process all raw sequences from the samples. This process included quality control (filtered), denoising (correcting sequencing errors), merging (for paired-end data), and removing chimeric sequences to form operational taxonomic units (OTUs). To study the species composition and diversity information of the samples, representative OTU sequences were selected and compared against databases (the Greengenes Database version 13_8 for 16S, the Silva release 132 for 18S, and the UNITE database version 8.2 for ITS) to obtain species annotation information. Based on the absolute abundance and annotation information of OTUs, the proportion of sequences at seven taxonomic levels (Kingdom, Phylum, Class, Order, Family, Genus, Species) for each sample was calculated. For comparative analysis between samples, Venn diagrams were used to identify unique or shared OTUs based on their presence in different sample groups. LEfSe analysis was employed to identify significant differences in species at various taxonomic levels between groups. Alpha and beta diversity analyses were primarily conducted using the Qiime2 diversity plugin. The phylogenetic tree was constructed using the “ggtree” package in R, with a focus on the representative OTU sequences. The most abundant OTU for each genus and the top 50 genera based on abundance were selected for phylogenetic analysis. This evolutionary relationship was visualized with a phylogenetic tree, and heatmaps were used to display the absolute abundance of OTUs across different groups. Through bioinformatics analysis, we obtained a wealth of information on the composition, abundance, phylogenetics, and community comparisons of bacterial and archaeal species in specific experimental samples.

### Statistical analyses

The primary analysis for the MR study employed the inverse-variance weighted (IVW) method, the weighted median estimator, the simple mode, the weighted mode, and the MR-Egger method. All MR analyses were conducted using R software (version 4.3.0) and utilized the “MendelianRandomization” and “MRPRESSO” packages. The LEfSe method was used to perform a differential abundance analysis based on the relative abundance of the microbiota. A Venn diagram analysis was used to identify unique or shared operational taxonomic units (OTUs) between the two groups, and the number of shared and unique OTUs in the samples was calculated. Alpha- and beta-diversity analyses were performed using the qiime2 diversity plugin. After obtaining the general alpha diversity indices, the Kruskal–Wallis test was used to compare whether there were significant differences in the alpha diversity indices between different sample groups, taking into account the grouping information. Principal coordinates analysis was conducted based on the Unweighted UniFrac distance, and the principal coordinate combination with the highest contribution rate was selected for visualization. The evolutionary tree was constructed using the “ggtree” package in the R programming language. The selected representative OTU sequences were used for phylogenetic analysis. Support vector machine and random forest model analysis were performed with the R environment using the “glmne,” “caret,” and “randomForest” package.

### Ethical approval

All selected GWAS for the gut microbiota of the MiBioGen consortium and AP from the FinnGen consortium were ethically approved and informed consent was provided from the individuals involved. As the original GWAS had previously received appropriate ethics and institutional review board approval, ethical approval for their use was not required for this study.

In the nested case–control study, the clinical data of patients with AP admitted to the general surgery department of Xuanwu Hospital of Capital Medical University between 1 January 2014, and 31 December 2020, were analyzed. This study was reviewed and approved by the Ethics Review Committee of the Xuanwu Hospital of Capital Medical University (No. 2020158). The study was designed in accordance with the principles of the Declaration of Helsinki (revised in 2013). To further ensure the protection of participants’ rights and privacy, we implemented stringent measures for handling patient data, especially genetic data. All data were anonymized and securely stored, and access was restricted to authorized personnel only. We adhered to best practices for data security and confidentiality throughout the study, including data encryption and secure data transfer protocols. Additionally, participants were fully informed about the scope and purpose of the study, and their consent included permission for the use of their genetic data in this research. These measures were taken to mitigate any potential risks associated with the use of sensitive genetic information.

All sequence data associated with this project have been deposited in the NCBI Short.

Read Archive database (accession number: PRJNA 1117862).

## Results

### Bidirectional Mendelian randomization study of gut microbiota and acute pancreatitis

**Figure 1:**
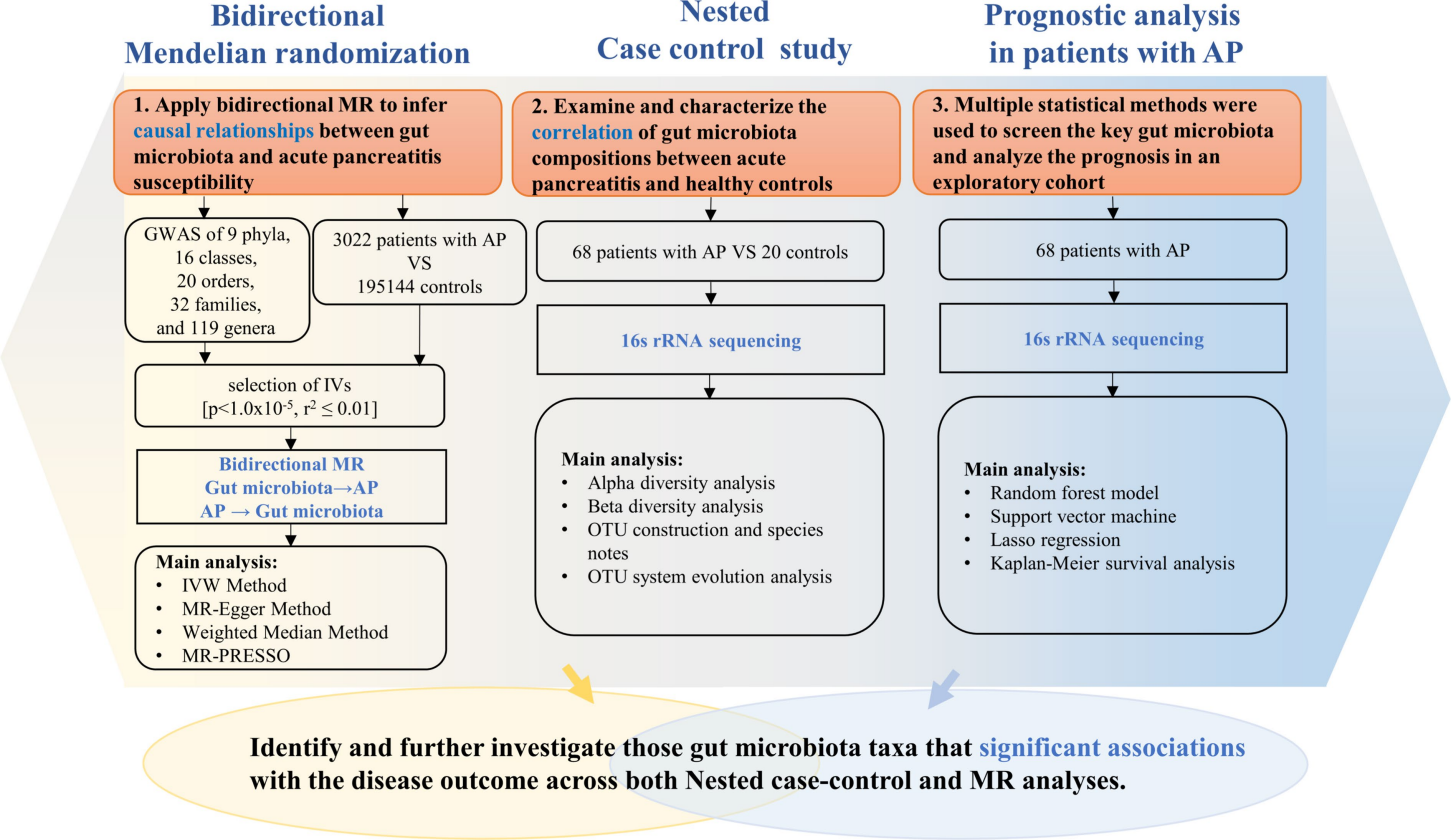
Description of the study design and analysis process.

**Figure 2:**
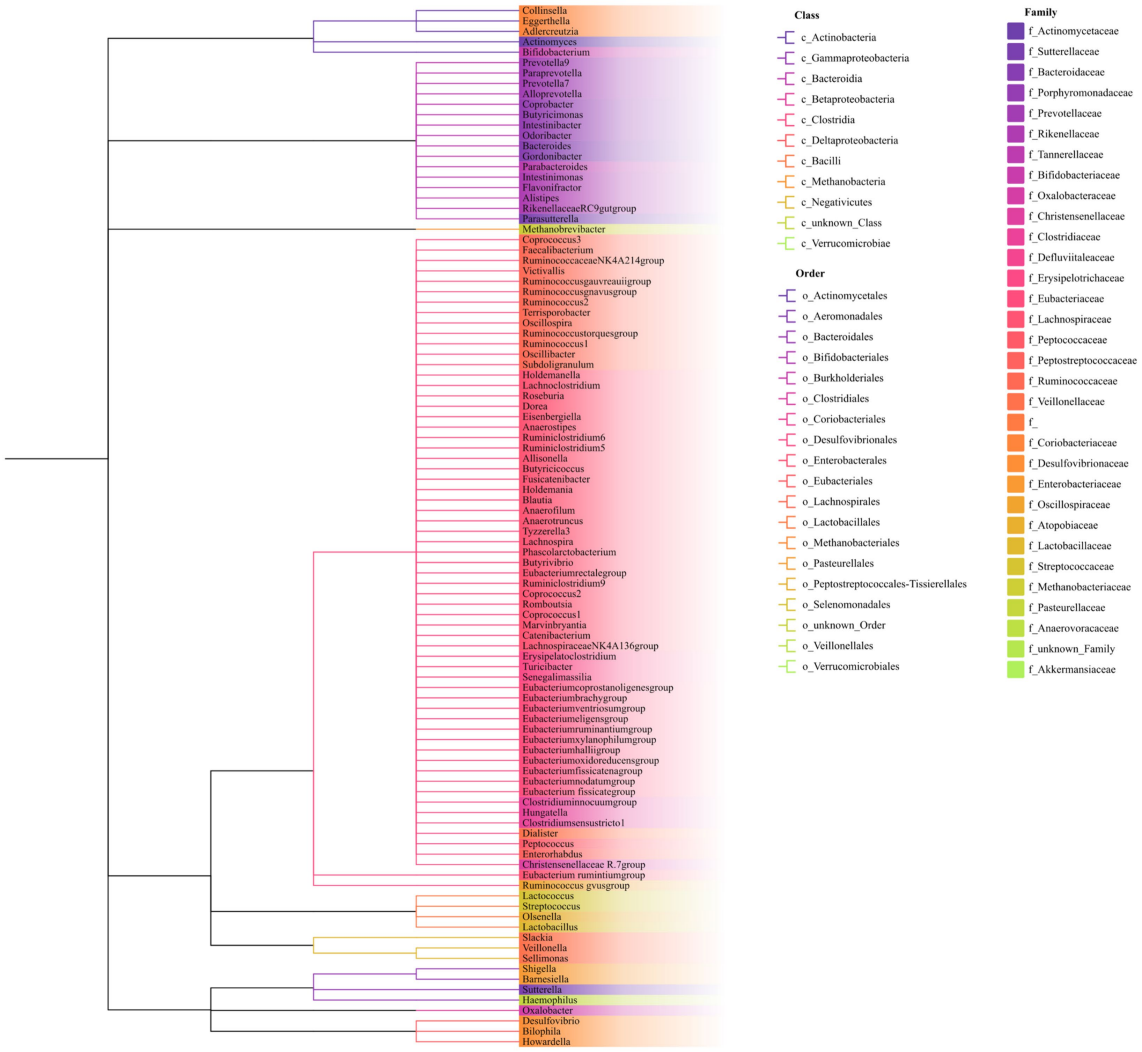
Phylogenetic tree of the gut microbiota. The phylogenetic tree includes nine phyla, 16 classes, 20 orders, 32 families, and 119 genera. Genus names are color-coded according to their respective families. Classes and orders are separated and identified by distinct branch patterns within the tree.

As shown in Figure 1, our study design offers a comprehensive blueprint to unravel the genetic causal link between the gut microbiota and AP. We applied a bidirectional causal correlation encompassing various bacterial characteristics (9 phyla, 16 classes, 20 orders, 32 families, and 119 genera) as shown in Figure 2. This approach enabled the identification of specific members of the gut microbiota showing a causal association with AP.

Following the PLINK clumping procedure for LD, we shortlisted 2,953 SNPs associated with gut microbiota characteristics (*p* < 1 × 10^−5^) to serve as IVs. These variables span five hierarchical strata: phylum, class, order, family, and genus. Given that lower-level microbiota traits may correlate with higher-tier traits, there is a potential overlap among the selected SNPs. The F-statistics for these SNPs surpassed the threshold of 10, alluding to a reduced propensity for weak instrument bias in our causal estimates (Supplementary Table S1). Procedures for proxy SNP discovery and palindromic SNP exclusion were conducted concurrently.

**Figure 3:**
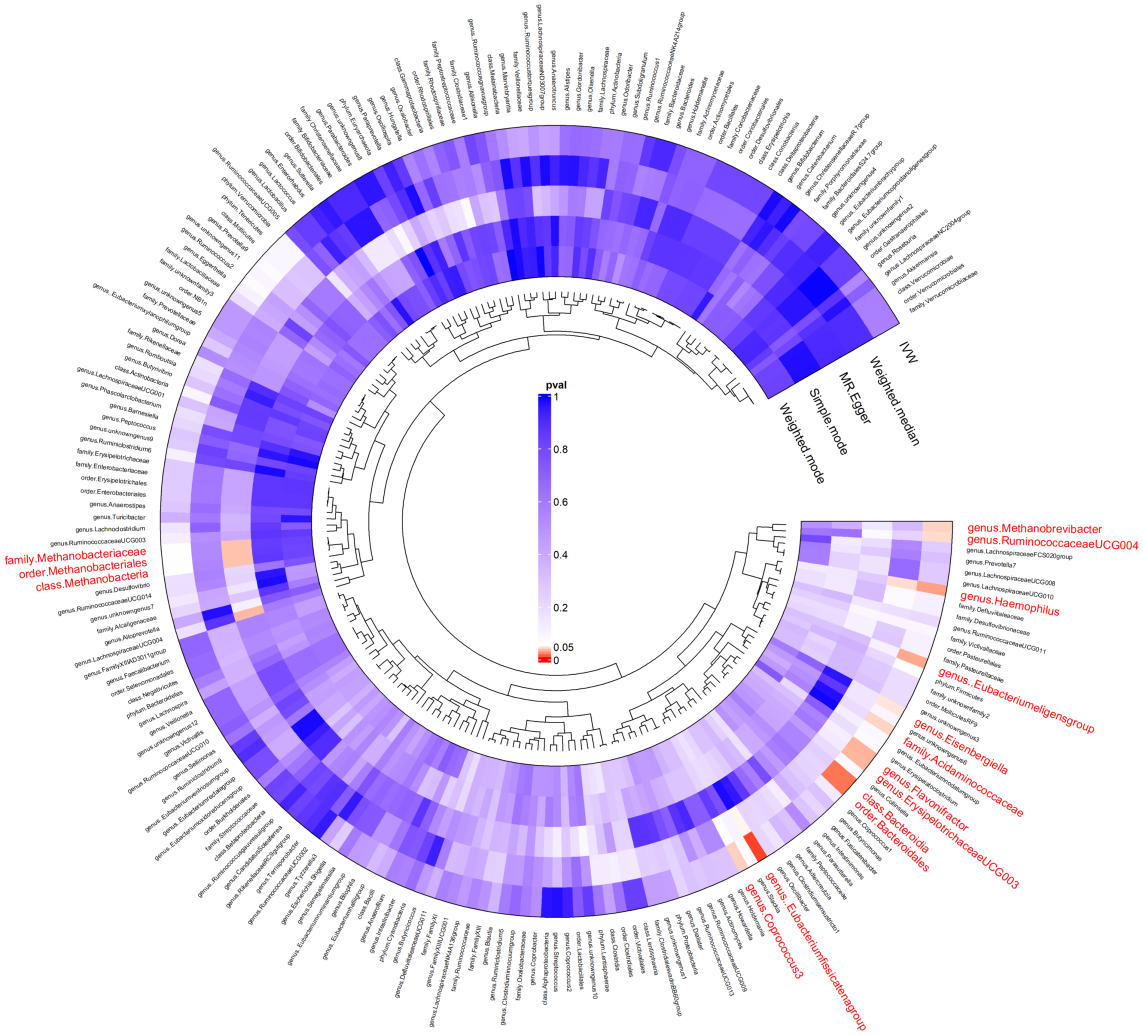
Results of bidirectional Mendelian randomisation of gut microbiota and acute pancreatitis. Ring clustering heatmap plot of the significant inverse-variance weighted (IVW) results of the gut microbiota on acute pancreatitis.

In our forward MR analysis (Figure 3), we aimed to understand the intricate relationship between the gut microbiota and AP. Our significant IVW results provided clear insights (Supplementary Figure S1). In particular, we discovered that microbes, such as *c_Methanobacteria* and *f_Methanobacteriaceae*, when genetically predicted at higher concentrations, were associated with a decreased risk of AP, suggesting the potential of certain microbial taxa to mitigate the risks associated with pancreatic inflammation and tissue damage by regulating gut metabolism, producing beneficial metabolites, and fostering gut health. Conversely, groups such as *c_Bacteroidia* and *o_Bacteroidales* were positively correlated with an increased risk of AP. These microbial groups can exert their effects by releasing harmful metabolites, impairing the intestinal barrier, inducing inflammatory reactions, or modulating immune responses.

In our reverse MR assessment (Supplementary Figure S2), we examined the relationship between AP and gut microbiota (Supplementary Figure S3). We found that a higher genetically predicted risk of AP was associated with lower concentrations of *o_Bacillales* and *g_Candidatus soleaferrea* and higher concentrations of *f_Bacteroidaceae* and *g_Bacteroides*.

Weighted median and MR-Egger methods supported the association findings. The MR-Egger regression intercept did not reveal evidence of horizontal pleiotropy (all intercepts, *p* > 0.05), suggesting the absence of unidirectional pleiotropic drifts. The Cochran Q test did not indicate any heterogeneity (*p* > 0.05) and the global MR-PRESSO test did not reveal any indication of pleiotropic effects (*p* > 0.05). For a more granular understanding, Supplementary Table S2 catalogues the relationship between the gut microbiota and AP.

### Nested case–control study

**Figure 4:**
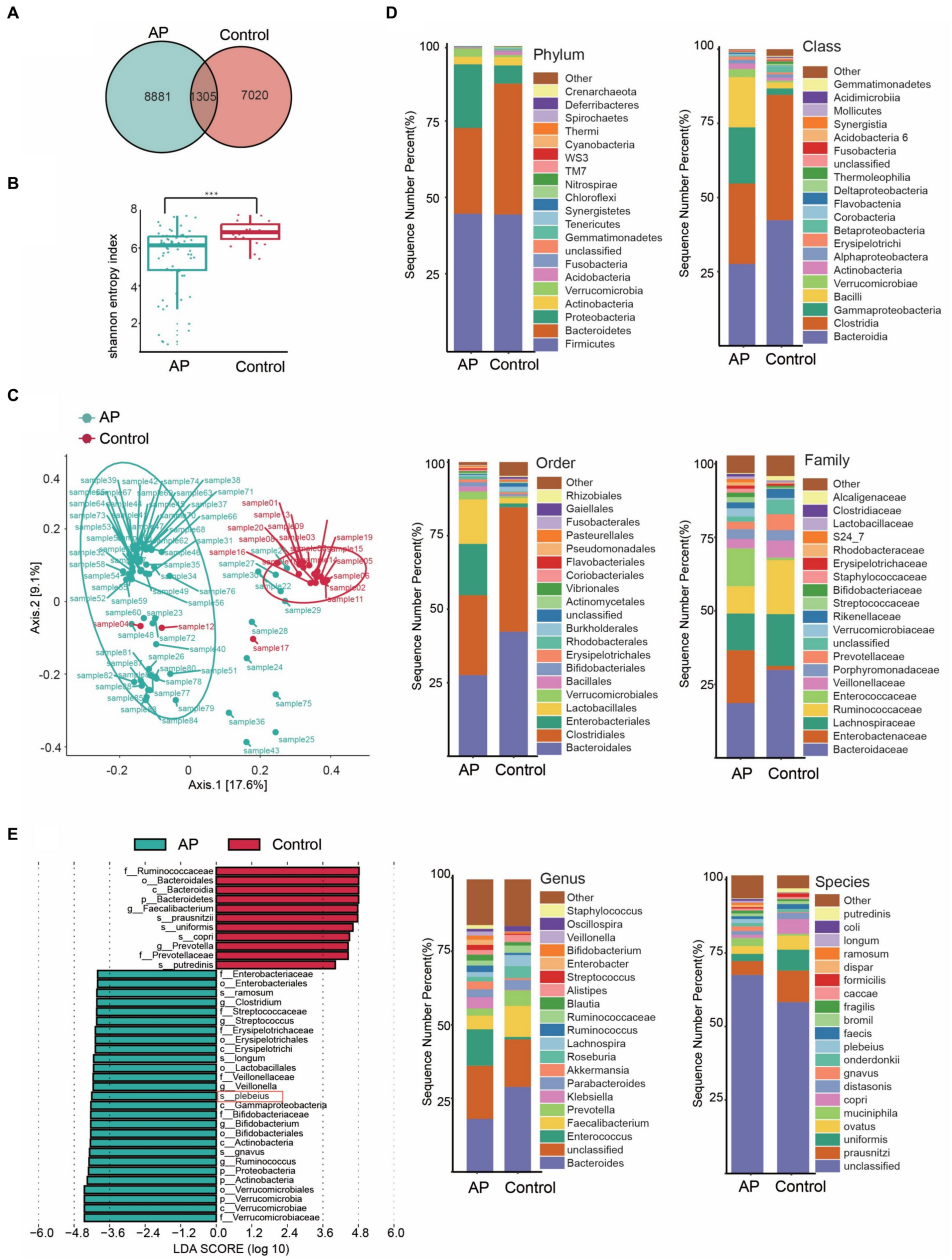
Characteristics of the gut microbiota between case and control. **(A)** Venn diagram of common or endemic species between cases and controls. **(B)** Testing for difference between case and control in Alpha diversity index. **(C)** Principal coordinate analysis of Beta diversity. **(D)** Bar graph of the relative distribution of cases and controls at each level (relative abundance of the top 20 species). **(E)** Linear discriminant analysis (LDA) bar plots of effect size (LEfSe) analysis, each transverse column represents a strain, and the length of the column corresponds to the LDA value.

Several studies have observed increased abundances of *Bacteroidia* in faecal samples from patients with AP before treatment, suggesting that this genus may be involved in the onset and development of AP, which is consistent with the results of our bi-directional MR analysis. To analyze the difference of gut microbiota between patients with AP and controls and the effect of the key species on the rehabilitation prognosis of patients with AP, we designed a nested case–control study to verify the MR findings. To this end, faecal samples were collected from 88 participants: 68 patients and 20 healthy controls. A Venn diagram shows the differences in bacterial species between the two groups (Figure 4A).

Diversity is an essential metric for understanding microbial populations. Alpha diversity (the Shannon index) and beta diversity provide critical insights into this aspect. Our findings revealed a reduced diversity of the gut microbiota in patients with AP (Figure 4B). This was evident when comparing the Shannon index between the healthy group and the patients with AP (*p* < 0.001; Supplementary Table S1). For the assessment of beta diversity, we employed principal coordinates analysis based on the weighted UniFrac distance, which demonstrated a pronounced difference in gut microbiota composition between the groups (*p* < 0.001) (Figure 4C).

To obtain a deeper understanding of the dynamics of the microbial composition during the disease process, we analyzed the relative distribution of microbial samples at various taxonomic levels. The bar graph in Figure 4D visually illustrates these disparities between the groups, highlighting significant variations. Figure 4E shows the species that differed the most in OTU abundance between the groups.

Our analysis focused on the most abundant species (Figure 4E) highlighted the dominance of certain species, such as *s_prausnitzii, s_uniformis* and *s_copri* in the control group. In stark contrast, the group encompassing patients with AP prominently showed species such as *s_gnavus*, *s_plebeius* and *s_longum*. Intriguingly, the abundance of *Bacteroides* taxa, such as *Bacteroides plebeius,* increased in patients.

A gut microbiota-based signature can predict the status of acute pancreatitis patients.

**Figure 5:**
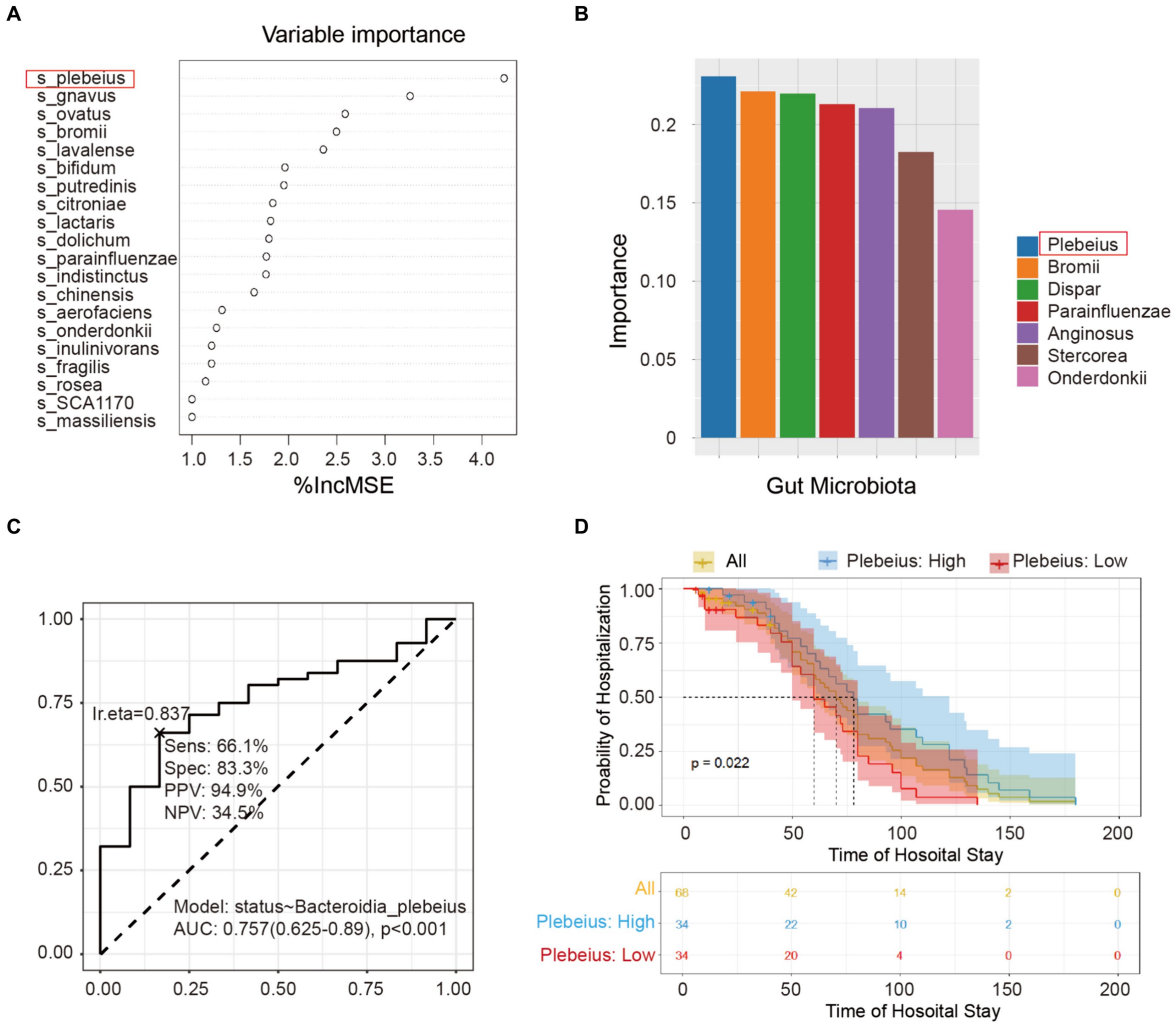
Screening the key gut microbiota related to the prognosis of acute pancreatitis using multiple models. **(A)** Variable importance plot from random forest analysis. Variables are organised in order of importance, where the strongest predictors are given at the top of each list. **(B)** Variable importance plot from the support vector machine. The variables were ordered in order of importance, with the strongest predictors placed to the left. **(C)** Receiver operating characteristic (ROC) curve of *Bacteroidia plebeius*. **(D)** Kaplan–Meier survival analysis of *B. plebeius* in patients with AP after treatment.

To identify the pivotal species that affect the prognosis of patients with AP, we used two sophisticated statistical approaches, including RF and SVM. Figure 5A delineates the importance of the variables derived from the random forest model, while Figure 5B illustrates the significance scores of the variables in the SVM. After a comprehensive evaluation of the results of these two models, the key intestinal microbe *B. plebeius* was found to play a significant role in influencing the hospital status. By directing our focus toward the prognostic analysis, we examined the correlation between *B. plebeius* abundance and the incidence of AP. Using a receiver operating characteristic (ROC) curve, we determined the predictive power of *B. plebeius*, which revealed a promising area under the curve (AUC) of 0.757 (Figure 5C). Kaplan–Meier survival analysis offered further insight into the prognostic implications of the abundance of *B. plebeius* in patients with AP. In particular, patients with an elevated abundance of *B. plebeius* experienced a prolonged hospital stay, as shown in Figure 5D (*p* < 0.05). These findings highlight the importance of specific gut microbiota, especially, *B. plebeius*, in influencing the progression and prognosis of patients with AP.

## Discussion

In the present study, we found that genetic susceptibility and an increased relative abundance of *B. plebeius* were associated with an increased risk of AP. Historically, the link between the gut microbiota and AP has been nebulous and inconclusive (Patel et al., 2021). In this study, we used a combination of bidirectional MR analysis and a nested case–control study to explore the relationship between the gut microbiota and AP.

Using bidirectional MR, we identified specific gut microbial communities with a causal relationship with AP. We found that the genetic susceptibilities of 15 microbial taxa were associated with the risk of AP. These microbial taxa may contribute to the risk through the production of harmful metabolites, impaired intestinal barrier function, induction of inflammatory responses, or modulation of immune regulation (Sun et al., 2019; Wei et al., 2019; Di Vincenzo et al., 2024). Some microbial communities can also reduce the risk of pancreatitis-related inflammation and tissue damage by regulating intestinal metabolism, producing beneficial metabolites, and promoting intestinal health (Zhang et al., 2019; Tegegne and Kebede, 2022). Genetic susceptibility to AP was correlated with the abundances of eight microbial taxa. AP may affect the composition and function of gut microbial communities through pathways including, but not limited to, inflammation, immune regulation, and disruption of intestinal barrier function (Li et al., 2020; Wang et al., 2022). To the best of our knowledge, this is the first study to apply MR to explore the causal relationship between the gut microbiome and AP. Although disease phenotypes in a population can be influenced by genetic and environmental factors, the direct use of genetically correlated GWAS data and rigorous quality control of the included SNPs can help avoid confounding factors and reverse causality effects.

In the MR analysis, we analyzed SNPs, which are indicators of genetic susceptibility, and their proximal gene functions (Supplementary Table S3). We hypothesised that their encoded proteins may influence the gut microbial community through different mechanisms. These mechanisms include the regulation of gene transcription (Liu et al., 2021), cell division (Kee et al., 2012), cell signaling (Perissinotti et al., 2014), fatty acylation (Niu et al., 2019), immune modulation (Wilson et al., 2012), cell membrane genesis (Raghu et al., 2021), and protein degradation (Castorena-Torres et al., 2008). Our detailed investigation of SNPs representing genetic susceptibility to AP (Supplementary Table S3) revealed their association with digestive enzyme activity (Bertin et al., 2023), cell signaling and cycle regulation (Pelech and Sanghera, 1992), immune regulation and immune response (Masson et al., 2023), cholesterol and plant steroid transport (Yu et al., 2014), neural development and neural function (de Castro-Catala et al., 2017), ion channels and regulation of cell excitability (Al-Bataineh et al., 2023), cell adhesion and migration (Camacho Leal et al., 2018), pancreatic function and trypsin regulation (LaRusch et al., 2015), REDOX reactions, and cellular stress response (Janda et al., 2015). Functional changes influenced by these SNPs may modulate the metabolism, growth, and interactions of microorganisms. However, empirical evidence to support this causal relationship is lacking, emphasizing the need for further research.

Exploring the interplay between these proteins and the gut microbiota and AP can elucidate their regulatory roles within the microbial framework. These taxa exhibited a hierarchical relationship, indicating potential causal interactions between the different *Bacteroides* genera and AP. These microbes not only increase the risk of AP development but also promote disease progression once AP occurs, thus forming a vicious cycle that contributes to the rapid progression of AP. These dynamic changes provide insight into AP pathogenesis. This study could pave the way for the development of innovative prevention and treatment strategies. However, these hypotheses must be validated through rigorous experimental studies. After confirming the causal relationship between the gut microbiome and AP through bidirectional MR analysis, we validated the results through a nested case–control study. We successfully verified the differences in the relative abundance of various gut microbial taxa between the healthy control group and patients with AP. In particular, therapeutic interventions for AP may achieve efficacy by modulating the abundance of *B. plebeius*, which was universally increased in the patient cohort.

Although research on the relationship between the gut microbiome and AP is limited, existing studies have consistently reported a decrease in microbial diversity among patients with AP, which aligns with the findings of our nested case–control study. However, there may have been variations in the identification of specific microbial taxa. For example, Zou et al. (2022) observed higher abundances of *p_Bacteroidetes, o_Bacteroidales, f_Bacteroidaceae, and g_Bacteroides* in stool samples from patients with AP than in a control group. This is partially consistent with the results of Zhang et al. (2018), suggesting that therapeutic interventions may achieve efficacy by modulating the abundance of members from Bacteroidetes. These differences in results could be attributed to several factors. Firstly, variations in study design, such as differences in sample collection, sequencing techniques, and analysis methods, can significantly impact findings. Additionally, demographic and clinical characteristics of the study populations, including age, diet, genetic background, and geographic location, can influence gut microbiota composition. Smaller sample sizes in some studies may also contribute to variability in outcomes. Environmental exposures and lifestyle factors, such as antibiotic usage, diet, and overall health, further affect the gut microbiota, leading to different results across studies. Technical variability, including differences in sequencing platforms and bioinformatics pipelines, can also introduce inconsistencies. Moreover, the timing of sample collection relative to the stage of AP and the analytical approaches used for data analysis can influence the identification of significant microbial taxa. Standardizing these aspects across studies is crucial to reduce discrepancies and achieve more consistent results. To study the difference and the key gut microbiota between AP cases and controls, we analyzed faecal samples using 16S rRNA gene sequencing in our nested case–control study and performed follow-up examinations on the included patients with AP. We employed multiple statistical models, including RF and SVM, to identify gut microbial taxa that may play a crucial role in the prognosis of patients with AP. We analyzed the relationship between *B. plebeius* and patient survival outcomes. The analysis of the ROC curve showed that the AUC for *B. plebeius* was 0.757, indicating its potential as a biomarker to predict the prognosis of patients with AP. Kaplan–Meier survival analysis further confirmed the importance of *B. plebeius*. By integrating the results of the above analyses, we identified *B. plebeius* as a particularly influential group, a finding that is further supported by various study designs and statistical methods in this study.

Our findings indicate that patients with elevated abundances of *B. plebeius* had longer hospital stays, which emphasizes its importance in the dynamics of AP.

*Bacteroidia plebeius* regulates AP through its involvement in inflammation, immune regulation, and lipid metabolism. However, some studies suggest that *Bacteroidia* may produce anti-inflammatory agents, such as short-chain fatty acids, which can alleviate pancreatitis and injury (Fan et al., 2023). They can also interact with the host immune system to modulate immune cell activity and intensity of inflammatory responses, thus influencing the severity and prognosis of AP (Wexler, 2007).

Certain *Bacteroidia* species typically play a beneficial role in the gut, but can also act as opportunistic pathogens in other parts of the body (Zafar and Saier, 2021). Factors such as immunodeficiency, intestinal barrier disruption, surgical injury, excessive antibiotic use, ageing, and dietary patterns can lead to their translocation from the gut to other parts of the body. They can degrade mucus, leading to intestinal inflammation and compromised barrier function, thus allowing the spread of potential pathogens. In addition, *Bacteroidia* species can transfer virulence genes to neighboring cells, enhancing their pathogenicity. Initially, aerobic bacteria cause tissue damage; however, as oxygen levels decrease, anaerobic genera such as *Bacteroidia* proliferate, resulting in inflammation, diarrhoea, and the formation of abdominal abscesses. The dissemination of *Bacteroidia* species outside the gut can cause bacteraemia and abscess formation at different locations, including the central nervous system (Zafar and Saier, 2021). They may be involved in cholesterol metabolism and gallstone formation (Grigor’eva and Romanova, 2020), which are related to the pathogenesis of AP and should be considered. Similarly, *B. plebeius* is also associated with various diseases and infections, including multiple system atrophy (Wan et al., 2019) and diabetic kidney disease (He et al., 2022). However, the physiological function and pathogenic mechanism of *B. plebeius* need to be further studied.

Despite these insights, the exact mechanisms underlying the relationship between the gut microbiota and AP require comprehensive explorations. The gut microbiota represents a multi-faceted ecosystem, and its interaction with AP can be influenced by a variety of factors and pathways. Although these findings are promising, they require further investigation. It is crucial to determine whether the increase in the abundance of *B. plebeius* in patients with AP is the cause or consequence of a prolonged hospital stay. Is it possible that treatments, dietary changes, or inflammation associated with AP directly affect the abundance of *B. plebeius*? Future studies should focus on elucidating the potential mechanisms underlying these phenomena to pave the way for microbial-based therapeutic strategies against AP. Further studies should go deeper into these mechanisms and validate the roles of the gut microbiota in the occurrence and development of AP.

Our study has several strengths. First, its multi-layered and multi-faceted study design integrated MR, nested case–control studies, and prognostic analysis of AP cases, which enhanced the credibility of our research findings. Furthermore, MR uses exposure and outcome datasets derived from large-scale GWAS or GWAS meta-analyses. The substantial F-statistics of our genetic instruments (see Supplementary Tables S3) indicated minimal potential for weak instrument bias. However, this study had a few limitations. In MR, caution should be exercised when interpreting SNPs as surrogate indicators of exposure, particularly when extrapolating genetic susceptibility to AP or the abundance of specific members of the gut microbiota. The occurrence of AP and the abundance of gut microbiota are influenced by multiple factors, with host genetics being one aspect. Additionally, nested case–control studies are cross-sectional in nature and their inherent limitation is that they only describe the microbial distribution at the time of sampling, making causal inference challenging. Therefore, our robust conclusions stem from the triangulation of MR, nested case–control studies, and prognostic analysis. Finally, the mechanisms underlying the relationship between the gut microbiota and AP remain unexplored, necessitating further comprehensive research. To address these limitations, future investigation should aim to include longitudinal studies and experimental models to validate our findings and further elucidate the complex interactions between the gut microbiota and AP.

## Conclusion

By combining MR with a nested case–control study, our research provides a detailed characterization of the interactions between the gut microbiota and AP. We identified *B. plebeius* as an important contributor, revealing its potential as a precursor and consequence of the dynamics of AP. Furthermore, our insights into changes in the gut microbiota emphasize the multi-factorial nature of AP and its complex relationship with the internal microbial ecosystem. This study lays the foundation for the future exploration of innovative therapeutic interventions that target these microbial dynamics in the treatment of AP.

## Data availability statement

Publicly available datasets were analyzed in this study. This data can be found here: https://risteys.finregistry.fi/endpoints/K11_ACUTPANC. All sequence data associated with this project have been deposited in the NCBI Short Read Archive database (accession number: PRJNA 1117862).

## Ethics statement

All selected GWAS for the gut microbiota of the MiBioGen consortium and acute pancreatitis (AP) from the FinnGen consortium were ethically approved, and informed consent was provided by the individuals involved. As the original GWAS had previously received appropriate ethics and institutional review board approval, ethical approval was not required for this study. This study was reviewed and approved by the Ethics Review Committee of the Xuanwu Hospital of Capital Medical University (No. 2020158). The study was designed in accordance with the principles of the Declaration of Helsinki (revised in 2013).

## Author contributions

CQ: Writing – original draft, Writing – review & editing. JLu: Writing – original draft, Writing – review & editing. YC: Writing – original draft, Writing – review & editing. JLi: Writing – original draft, Writing – review & editing. XX: Writing – original draft, Writing – review & editing. FL: Writing – original draft, Writing – review & editing.

## Footnotes

^1^ https://github.com/cyyyyyyyyy666/Code_for_Article_Data_analysis/tree/master

## Supplementary material

SUPPLEMENTARY FIGURE S1 Forward MR assessment exploring the relationship between AP and gut microbiota.

SUPPLEMENTARY FIGURE S2 Reverse MR assessment exploring the relationship between AP and gut microbiota.

SUPPLEMENTARY FIGURE S3 Detailed visualization of the relationship between AP and gut microbiota from the reverse MR assessment.

SUPPLEMENTARY TABLE S1 Shannon index comparison between the healthy control group and patients with AP.

SUPPLEMENTARY TABLE S2 Catalog of the relationship between the gut microbiota and AP.

SUPPLEMENTARY TABLE S3 Analysis of SNPs as indicators of genetic susceptibility.

## References

[ref1] Al-Bataineh M. M. Kinlough C. L. Marciszyn A. Lam T. Ye L. Kidd K.. (2023). Influence of glycoprotein MUC1 on trafficking of the Ca^2+^-selective ion channels, TRPV5 and TRPV6, and on in vivo calcium homeostasis. J. Biol. Chem. 299:102925. doi: 10.1016/j.jbc.2023.102925, PMID: 36682497 PMC9996365

[ref2] Bäckhed F. Ley R. E. Sonnenburg J. L. Peterson D. A. Gordon J. I. (2005). Host-bacterial mutualism in the human intestine. Science 307, 1915–1920. doi: 10.1126/science.1104816, PMID: 15790844

[ref3] Bertin N. Tan J. Li Z. Gonzalez-Porta M. Rajaby R. Jimenez R.. A catalogue of structural variation across ancestrally diverse Asian genomes. (2023). [Preprint] https://www.researchsquare.com/article/rs-3376868/v1 (Accessed October 3, 2023). 10.1038/s41467-024-53620-8 PMC11535549 39496583

[ref4] Cabreiro F. Au C. Leung K. Y. Vergara-Irigaray N. Cochemé H. M. Noori T.. (2013). Metformin retards aging in *C. elegans* by altering microbial folate and methionine metabolism. Cell 153, 228–239. doi: 10.1016/j.cell.2013.02.035, PMID: 23540700 PMC3898468

[ref5] Camacho Leal M. D. P. Costamagna A. Tassone B. Saoncella S. Simoni M. Natalini D.. (2018). Conditional ablation of p130Cas/BCAR1 adaptor protein impairs epidermal homeostasis by altering cell adhesion and differentiation. Cell Commun. Signal 16:73. doi: 10.1186/s12964-018-0289-z, PMID: 30390666 PMC6215608

[ref6] Castorena-Torres F. Bermúdez de León M. Cisneros B. Zapata-Pérez O. Salinas J. E. Albores A. (2008). Changes in gene expression induced by polycyclic aromatic hydrocarbons in the human cell lines HepG2 and A549. Toxicol. In Vitro 22, 411–421. doi: 10.1016/j.tiv.2007.10.009, PMID: 18494146

[ref7] de Castro-Catala M. Mora-Solano A. Kwapil T. R. Cristóbal-Narváez P. Sheinbaum T. Racioppi A.. (2017). The genome-wide associated candidate gene ZNF804A and psychosis-proneness: evidence of sex-modulated association. PLoS One 12:e0185072. doi: 10.1371/journal.pone.0185072 28931092 PMC5607189

[ref8] Di Vincenzo F. Del Gaudio A. Petito V. Lopetuso L. R. Scaldaferri F. (2024). Gut microbiota, intestinal permeability, and systemic inflammation: a narrative review. Intern. Emerg. Med. 19, 275–293. doi: 10.1007/s11739-023-03374-w, PMID: 37505311 PMC10954893

[ref9] Fan Y. Ju T. Bhardwaj T. Korver D. R. Willing B. P. (2023). Week-old chicks with high Bacteroides abundance have increased short-chain fatty acids and reduced markers of gut inflammation. Microbiol. Spectr. 11, e03616–e03622. doi: 10.1128/spectrum.03616-22 36719194 PMC10100795

[ref10] Grigor’eva I. N. Romanova T. I. (2020). Gallstone disease and microbiome. Microorganisms 8:835. doi: 10.3390/microorganisms8060835, PMID: 32498344 PMC7356158

[ref11] He X. Sun J. Liu C. Yu X. Li H. Zhang W.. (2022). Compositional alterations of gut microbiota in patients with diabetic kidney disease and type 2 diabetes mellitus. Diabetes Metab. Syndr. Obes. 15, 755–765. doi: 10.2147/DMSO.S347805, PMID: 35280499 PMC8911313

[ref12] Iyer S. Bawa E. P. Tarique M. Dudeja V. (2020). Know thy enemy-understanding the role of inflammation in severe acute pancreatitis. Gastroenterology 158, 46–48. doi: 10.1053/j.gastro.2019.11.039, PMID: 31770524

[ref13] Janda E. Lascala A. Carresi C. Parafati M. Aprigliano S. Russo V.. (2015). Parkinsonian toxin-induced oxidative stress inhibits basal autophagy in astrocytes via NQO2/quinone oxidoreductase 2: implications for neuroprotection. Autophagy 11, 1063–1080. doi: 10.1080/15548627.2015.1058683, PMID: 26046590 PMC4590600

[ref14] Kee Y. S. Ren Y. Dorfman D. Iijima M. Firtel R. Iglesias P. A.. (2012). A mechanosensory system governs myosin II accumulation in dividing cells. Mol. Biol. Cell 23, 1510–1523. doi: 10.1091/mbc.e11-07-0601, PMID: 22379107 PMC3327329

[ref15] Kurilshikov A. Medina-Gomez C. Bacigalupe R. Radjabzadeh D. Wang J. Demirkan A.. (2021). Large-scale association analyses identify host factors influencing human gut microbiome composition. Nat. Genet. 53, 156–165. doi: 10.1038/s41588-020-00763-1, PMID: 33462485 PMC8515199

[ref16] LaRusch J. Lozano-Leon A. Stello K. Moore A. Muddana V. O'Connell M.. (2015). The common chymotrypsinogen C (CTRC) variant G60G (C. 180T) increases risk of chronic pancreatitis but not recurrent acute pancreatitis in a north American population. Clin. Transl. Gastroenterol. 6:e68. doi: 10.1038/ctg.2014.13, PMID: 25569187 PMC4418406

[ref17] Li X. Y. He C. Zhu Y. Lu N. H. (2020). Role of gut microbiota on intestinal barrier function in acute pancreatitis. World J. Gastroenterol. 26, 2187–2193. doi: 10.3748/wjg.v26.i18.2187, PMID: 32476785 PMC7235204

[ref18] Li D. Wang P. Wang P. Hu X. Chen F. (2016). The gut microbiota: a treasure for human health. Biotechnol. Adv. 34, 1210–1224. doi: 10.1016/j.biotechadv.2016.08.003, PMID: 27592384

[ref19] Liu J. Huang Z. Chen H. N. Qin S. Chen Y. Jiang J.. (2021). ZNF37A promotes tumor metastasis through transcriptional control of THSD4/TGF-β axis in colorectal cancer. Oncogene 40, 3394–3407. doi: 10.1038/s41388-021-01713-9, PMID: 33875786

[ref20] Masson E. Ewers M. Paliwal S. Kume K. Scotet V. Cooper D. N.. (2023). The PRSS3P2 and TRY7 deletion copy number variant modifies risk for chronic pancreatitis. Pancreatology 23, 48–56. doi: 10.1016/j.pan.2022.11.013, PMID: 36517351

[ref21] Nicolien J. S. Bakker O. J. Besselink M. G. Ahmed Ali U. Bollen T. L. Gooszen H. G.. (2019). Impact of characteristics of organ failure and infected necrosis on mortality in necrotising pancreatitis. Gut 68, 1044–1051. doi: 10.1136/gutjnl-2017-314657 29950344

[ref22] Niu J. Sun Y. Chen B. Zheng B. Jarugumilli G. K. Walker S. R.. (2019). Fatty acids and cancer-amplified ZDHHC19 promote STAT3 activation through S-palmitoylation. Nature 573, 139–143. doi: 10.1038/s41586-019-1511-x, PMID: 31462771 PMC6728214

[ref23] Patel B. K. Patel K. H. Bhatia M. Iyer S. G. Madhavan K. Moochhala S. M. (2021). Gut microbiome in acute pancreatitis: a review based on current literature. World J. Gastroenterol. 27, 5019–5036. doi: 10.3748/wjg.v27.i30.5019, PMID: 34497432 PMC8384740

[ref24] Pelech S. L. Sanghera J. S. (1992). Mitogen-activated protein kinases: versatile transducers for cell signaling. Trends Biochem. Sci. 17, 233–238. doi: 10.1016/S0968-0004(00)80005-5, PMID: 1323888

[ref25] Perissinotti P. P. Ethington E. G. Cribbs L. Koob M. D. Martin J. Piedras-Rentería E. S. (2014). Down-regulation of endogenous KLHL1 decreases voltage-gated calcium current density. Cell Calcium 55, 269–280. doi: 10.1016/j.ceca.2014.03.002, PMID: 24703904

[ref26] Qin J. Li R. Raes J. Arumugam M. Burgdorf K. S. Manichanh C.. (2010). A human gut microbial gene catalogue established by metagenomic sequencing. Nature 464, 59–65. doi: 10.1038/nature08821, PMID: 20203603 PMC3779803

[ref27] Raghu P. Basak B. Krishnan H. (2021). Emerging perspectives on multidomain phosphatidylinositol transfer proteins. Biochim. Biophys. Acta Mol. Cell Biol. Lipids 1866:158984. doi: 10.1016/j.bbalip.2021.158984, PMID: 34098114 PMC7611342

[ref28] Sanderson E. Glymour M. M. Holmes M. V. Kang H. Morrison J. Munafò M. R.. (2022). Mendelian randomization. Nat. Rev. Methods Primers 2:6. doi: 10.1038/s43586-021-00092-5, PMID: 37325194 PMC7614635

[ref29] Sun L. Jia H. Li J. Yu M. Yang Y. Tian D.. (2019). Cecal gut microbiota and metabolites might contribute to the severity of acute myocardial ischemia by impacting the intestinal permeability, oxidative stress, and energy metabolism. Front. Microbiol. 10:1745. doi: 10.3389/fmicb.2019.01745, PMID: 31428065 PMC6687875

[ref30] Tegegne B. A. Kebede B. (2022). Probiotics, their prophylactic and therapeutic applications in human health development: a review of the literature. Heliyon 8:e09725. doi: 10.1016/j.heliyon.2022.e09725, PMID: 35785237 PMC9240980

[ref31] van Sandwort H. C. Bakker O. J. Bollen T. L. Besselink M. G. Ahmed Ali U. Schrijver A. M.. (2011). A conservative and minimally invasive approach to necrotizing pancreatitis improves outcome. Gastroenterology 141, 1254–1263. doi: 10.1053/j.gastro.2011.06.073 21741922

[ref32] Wan L. Zhou X. Wang C. Chen Z. Peng H. Hou X.. (2019). Alterations of the gut microbiota in multiple system atrophy patients. Front. Neurosci. 13:1102. doi: 10.3389/fnins.2019.01102, PMID: 31680836 PMC6813281

[ref33] Wang Z. Guo M. Li J. Jiang C. Yang S. Zheng S.. (2023). Composition and functional profiles of gut microbiota reflect the treatment stage, severity, and etiology of acute pancreatitis. Microbiol. Spectr. 11:e0082923. doi: 10.1128/spectrum.00829-23, PMID: 37698429 PMC10580821

[ref34] Wang Z. Li F. Liu J. Luo Y. Guo H. Yang Q.. (2022). Intestinal microbiota - an unmissable bridge to severe acute pancreatitis-associated acute lung injury. Front. Immunol. 13:913178. doi: 10.3389/fimmu.2022.913178, PMID: 35774796 PMC9237221

[ref35] Wang Z. Liu J. Li F. Luo Y. Ge P. Zhang Y.. (2022). The gut-lung axis in severe acute pancreatitis-associated lung injury: the protection by the gut microbiota through short-chain fatty acids. Pharmacol. Res. 182:106321. doi: 10.1016/j.phrs.2022.106321, PMID: 35752356

[ref36] Wei M. Y. Shi S. Liang C. Meng Q. C. Hua J. Zhang Y. Y.. (2019). The microbiota and microbiome in pancreatic cancer: more influential than expected. Mol. Cancer 18:97. doi: 10.1186/s12943-019-1008-0, PMID: 31109338 PMC6526613

[ref37] Wexler H. M. (2007). Bacteroides: the good, the bad, and the nitty-gritty. Clin. Microbiol. Rev. 20, 593–621. doi: 10.1128/CMR.00008-07, PMID: 17934076 PMC2176045

[ref38] Wilson T. J. Fuchs A. Colonna M. (2012). Cutting edge: human FcRL4 and FcRL5 are receptors for IgA and IgG. J. Immunol. 188, 4741–4745. doi: 10.4049/jimmunol.1102651, PMID: 22491254 PMC3634363

[ref39] Xiao A. Y. Tan M. L. Wu L. M. Asrani V. M. Windsor J. A. Yadav D.. (2016). Global incidence and mortality of pancreatic diseases: a systematic review, meta-analysis, and meta-regression of population-based cohort studies. Lancet Gastroenterol. Hepatol. 1, 45–55. doi: 10.1016/S2468-1253(16)30004-8, PMID: 28404111

[ref40] Yatsunenko T. Rey F. E. Manary M. J. Trehan I. Dominguez-Bello M. G. Contreras M.. (2012). Human gut microbiome viewed across age and geography. Nature 486, 222–227. doi: 10.1038/nature11053, PMID: 22699611 PMC3376388

[ref41] Yu X. H. Qian K. Jiang N. Zheng X. L. Cayabyab F. S. Tang C. K. (2014). ABCG5/ABCG8 in cholesterol excretion and atherosclerosis. Clin. Chim. Acta 428, 82–88. doi: 10.1016/j.cca.2013.11.010 24252657

[ref42] Zafar H. Saier M. H. Jr. (2021). Gut Bacteroides species in health and disease. Gut Microbes 13, 1–20. doi: 10.1080/19490976.2020.1848158, PMID: 33535896 PMC7872030

[ref43] Zhang Z. Tang H. Chen P. Xie H. Tao Y. (2019). Demystifying the manipulation of host immunity, metabolism, and extraintestinal tumors by the gut microbiome. Signal Transduct. Target. Ther. 4:41. doi: 10.1038/s41392-019-0074-5, PMID: 31637019 PMC6799818

[ref44] Zhang X. M. Zhang Z. Y. Zhang C. H. Wu J. Wang Y. X. Zhang G. X. (2018). Intestinal microbial community differs between acute pancreatitis patients and healthy volunteers. Biomed. Environ. Sci. 31, 81–86. doi: 10.3967/bes2018.010, PMID: 29409589

[ref45] Zhao L. (2013). The gut microbiota and obesity: from correlation to causality. Nat. Rev. Microbiol. 11, 639–647. doi: 10.1038/nrmicro3089 23912213

[ref46] Zmora N. Suez J. Elinav E. (2019). You are what you eat: diet, health and the gut microbiota. Nat. Rev. Gastroenterol. Hepatol. 16, 35–56. doi: 10.1038/s41575-018-0061-2, PMID: 30262901

[ref47] Zou M. Yang Z. Fan Y. Gong L. Han Z. Ji L.. (2022). Gut microbiota on admission as predictive biomarker for acute necrotizing pancreatitis. Front. Immunol. 13:988326. doi: 10.3389/fimmu.2022.988326 36105818 PMC9466706

