# Supplementary material for: Unravelling the role of gut microbiota in acute pancreatitis: integrating Mendelian randomization with a nested case–control study
Source: Front Microbiol. 2024 Jul 3;15:1401056. doi: 10.3389/fmicb.2024.1401056 (PMC11253135; doi:10.3389/fmicb.2024.1401056)
Supplement: Supplementary file 1 [file Data_Sheet_1.ZIP › Supplementary Material/Additional file 1/Supplementary Table S1.docx]

**Table S1.**  **The outcomes of lasso regression**

|  | s1 | OR |
| --- | --- | --- |
| (Intercept) | 2.109582 | 8.244797 |
| Bacteria | 0 | 1 |
| Actinobacteria | 0 | 1 |
| Bacteroidetes | 0 | 1 |
| Firmicutes | 0 | 1 |
| Proteobacteria | 0 | 1 |
| Verrucomicrobia | 0 | 1 |
| unclassified | 0 | 1 |
| Tenericutes | 0 | 1 |
| Fusobacteria | 0 | 1 |
| Actinobacteria.1 | 0 | 1 |
| Bacteroidia | -1.99E-05 | 0.99998 |
| Bacilli | 0 | 1 |
| Clostridia | 0 | 1 |
| Erysipelotrichi | 0 | 1 |
| Gammaproteobacteria | 0 | 1 |
| Betaproteobacteria | 0 | 1 |
| unclassified.1 | 0 | 1 |
| Coriobacteriia | 0 | 1 |
| Verrucomicrobiae | 0 | 1 |
| Deltaproteobacteria | 0 | 1 |
| Alphaproteobacteria | 0 | 1 |
| Mollicutes | 0 | 1 |
| Fusobacteriia | 0 | 1 |
| Epsilonproteobacteria | 0 | 1 |
| Bifidobacteriales | 0 | 1 |
| Bacteroidales | -2.09E-19 | 1 |
| Lactobacillales | 0 | 1 |
| Clostridiales | 0 | 1 |
| Erysipelotrichales | 0 | 1 |
| Enterobacteriales | 0 | 1 |
| Actinomycetales | 0 | 1 |
| Burkholderiales | 0 | 1 |
| unclassified.2 | 0 | 1 |
| Coriobacteriales | 0 | 1 |
| Verrucomicrobiales | 0 | 1 |
| Pseudomonadales | 0 | 1 |
| Bacillales | 0 | 1 |
| Desulfovibrionales | 0 | 1 |
| Pasteurellales | 0 | 1 |
| Xanthomonadales | 0 | 1 |
| Fusobacteriales | 0 | 1 |
| Campylobacterales | 0 | 1 |
| Rhizobiales | 0 | 1 |
| Turicibacterales | 0 | 1 |
| Sphingomonadales | 0 | 1 |
| RF39 | 0 | 1 |
| Gemellales | 0 | 1 |
| unclassified.3 | 0 | 1 |
| gnavus | 0 | 1 |
| formicilis | 0 | 1 |
| longum | 0 | 1 |
| ovatus | 0 | 1 |
| uniformis | 0 | 1 |
| distasonis | 0 | 1 |
| dolichum | 0 | 1 |
| caccae | 0 | 1 |
| prausnitzii | 0 | 1 |
| ramosum | 0 | 1 |
| onderdonkii | 0 | 1 |
| fragilis | 0 | 1 |
| torques | 0 | 1 |
| bromii | 0 | 1 |
| muciniphila | 0 | 1 |
| lenta | 0 | 1 |
| infantis | 0 | 1 |
| symbiosum | 0 | 1 |
| faecis | 0 | 1 |
| aerofaciens | 0 | 1 |
| salivarius | 0 | 1 |
| obeum | 0 | 1 |
| dispar | 0 | 1 |
| longicatena | 0 | 1 |
| putredinis | 0 | 1 |
| parainfluenzae | 0 | 1 |
| plebeius | 0 | 1 |
| hathewayi | 0 | 1 |
| pullicaecorum | 0 | 1 |
| producta | 0 | 1 |
| eutactus | 0 | 1 |
| callidus | 0 | 1 |
| parvula | 0 | 1 |
| coli | 0 | 1 |
| copri | 0 | 1 |
| adolescentis | 0 | 1 |
| aldenense | 0 | 1 |
| catus | 0 | 1 |
| stercorea | 0 | 1 |
| finegoldii | 0 | 1 |
| celatum | 0 | 1 |
| breve | 0 | 1 |
| anginosus | 0 | 1 |
| Bifidobacteriaceae | 0 | 1 |
| Enterococcaceae | 0 | 1 |
| Streptococcaceae | 0 | 1 |
| Lachnospiraceae | 0 | 1 |
| Ruminococcaceae | 0 | 1 |
| Erysipelotrichaceae | 0 | 1 |
| Enterobacteriaceae | 0 | 1 |
| unclassified.4 | 0 | 1 |
| Bacteroidaceae | 0 | 1 |
| Porphyromonadaceae | 0 | 1 |
| Rikenellaceae | 0 | 1 |
| Veillonellaceae | 0 | 1 |
| Coriobacteriaceae | 0 | 1 |
| Clostridiaceae | 0 | 1 |
| Alcaligenaceae | 0 | 1 |
| Verrucomicrobiaceae | 0 | 1 |
| Lactobacillaceae | 0 | 1 |
| Peptostreptococcaceae | 0 | 1 |
| Prevotellaceae | -8.07E-05 | 0.999919 |
| Pseudomonadaceae | 0 | 1 |
| Odoribacteraceae | 0 | 1 |
| Desulfovibrionaceae | 0 | 1 |
| Pasteurellaceae | 0 | 1 |
| Paraprevotellaceae | 0 | 1 |
| Mogibacteriaceae | 0 | 1 |
| S24_7 | 0 | 1 |
| Eubacteriaceae | 0 | 1 |
| Actinomycetaceae | 0 | 1 |
| Barnesiellaceae | 0 | 1 |
| Christensenellaceae | 0 | 1 |
| Fusobacteriaceae | 0 | 1 |
| Tissierellaceae | 0 | 1 |
| Xanthomonadaceae | 0 | 1 |
| Micrococcaceae | 0 | 1 |
| Turicibacteraceae | 0 | 1 |
| Gemellaceae | 0 | 1 |
| Sporolactobacillaceae | 0 | 1 |
| Sphingomonadaceae | 0 | 1 |
| Bifidobacterium | 0 | 1 |
| Enterococcus | 0 | 1 |
| Streptococcus | 0 | 1 |
| Oscillospira | 0 | 1 |
| unclassified.5 | 0 | 1 |
| Bacteroides | 0 | 1 |
| Blautia | 0 | 1 |
| Ruminococcus | 0 | 1 |
| Gemmiger | 0 | 1 |
| Parabacteroides | 0 | 1 |
| Alistipes | -7.10E-06 | 0.999993 |
| Clostridium | 0 | 1 |
| Lachnospiraceae_Clostridium | 0 | 1 |
| Coprococcus | 0 | 1 |
| Ruminococcaceae_Ruminococcus | 0 | 1 |
| Erysipelotrichaceae_Clostridium | 0 | 1 |
| Eubacterium | 0 | 1 |
| Dorea | 0 | 1 |
| Faecalibacterium | 0 | 1 |
| Klebsiella | 0 | 1 |
| Lactobacillus | 0 | 1 |
| Dialister | 0 | 1 |
| Phascolarctobacterium | 0 | 1 |
| Enterobacter | 0 | 1 |
| Prevotellaceae_Prevotella | -1.23E-06 | 0.999999 |
| Eggerthella | 0 | 1 |
| Roseburia | 0 | 1 |
| Sutterella | 0 | 1 |
| Veillonella | 0 | 1 |
| Akkermansia | 0 | 1 |
| Lachnospira | 0 | 1 |
| Collinsella | 0 | 1 |
| Odoribacter | 0 | 1 |
| Haemophilus | 0 | 1 |
| Megasphaera | 0 | 1 |
| Anaerostipes | 0 | 1 |
| Pseudomonas | 0 | 1 |
| Butyricicoccus | 0 | 1 |
| Bilophila | 0 | 1 |
| Pseudoramibacter_Eubacterium | 0 | 1 |
| Butyricimonas | 0 | 1 |
| Escherichia | 0 | 1 |
| Actinomyces | 0 | 1 |
| Paraprevotella | 0 | 1 |
| Anaerotruncus | 0 | 1 |
| Peptostreptococcus | 0 | 1 |
| Turicibacter | 0 | 1 |
| Peptostreptococcaceae_Clostridium | 0 | 1 |
| Fusobacterium | 0 | 1 |
| Desulfovibrio | 0 | 1 |
| Rothia | 0 | 1 |
