# Supplementary figures and images for: Unravelling the role of gut microbiota in acute pancreatitis: integrating Mendelian randomization with a nested case–control study
Source: Front Microbiol. 2024 Jul 3;15:1401056. doi: 10.3389/fmicb.2024.1401056 (PMC11253135; doi:10.3389/fmicb.2024.1401056)

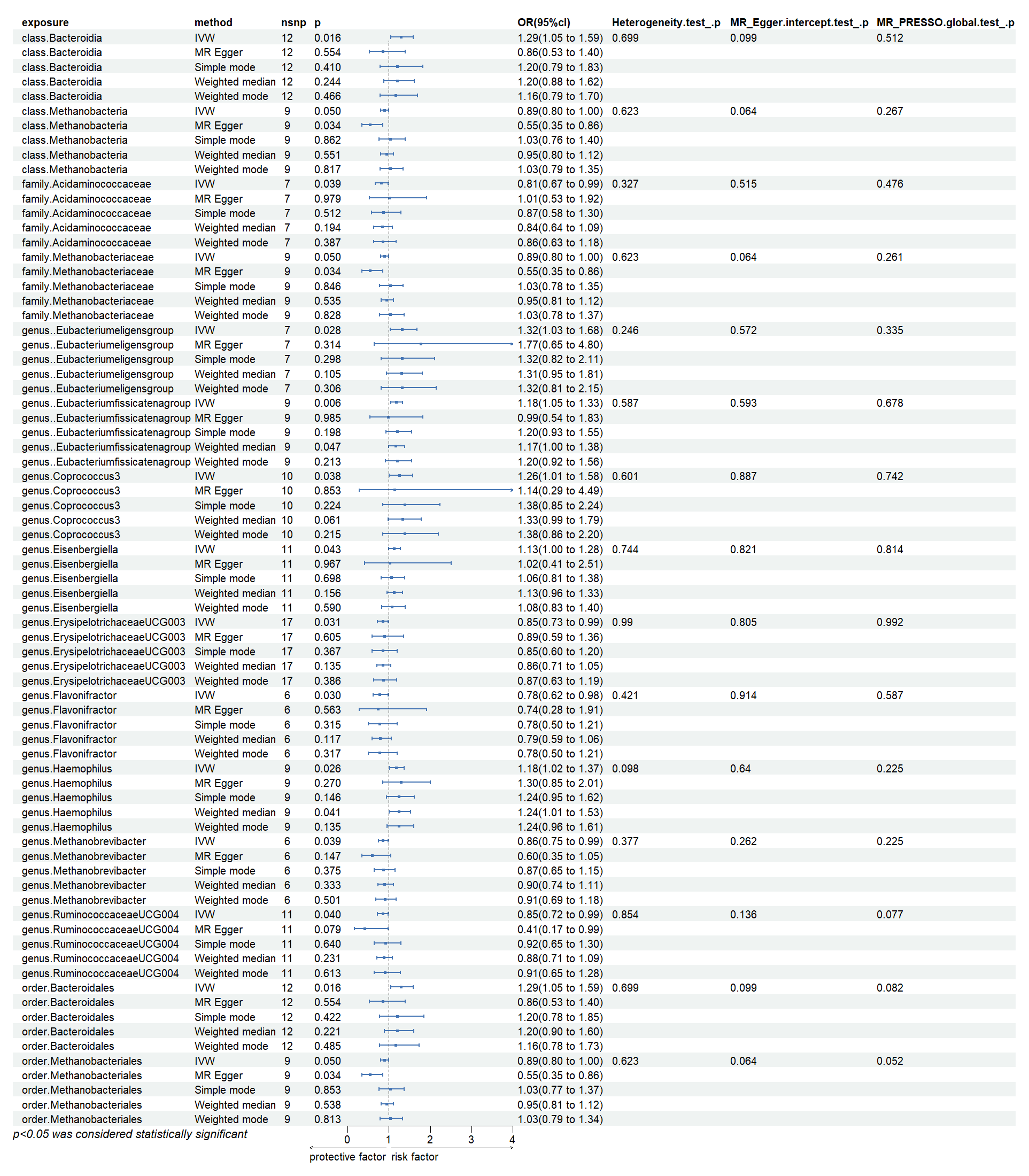

Supplement: Supplementary file 1 [file Data_Sheet_1.ZIP › Supplementary Material/Additional file 2/Supplementary Figure S1.tiff]

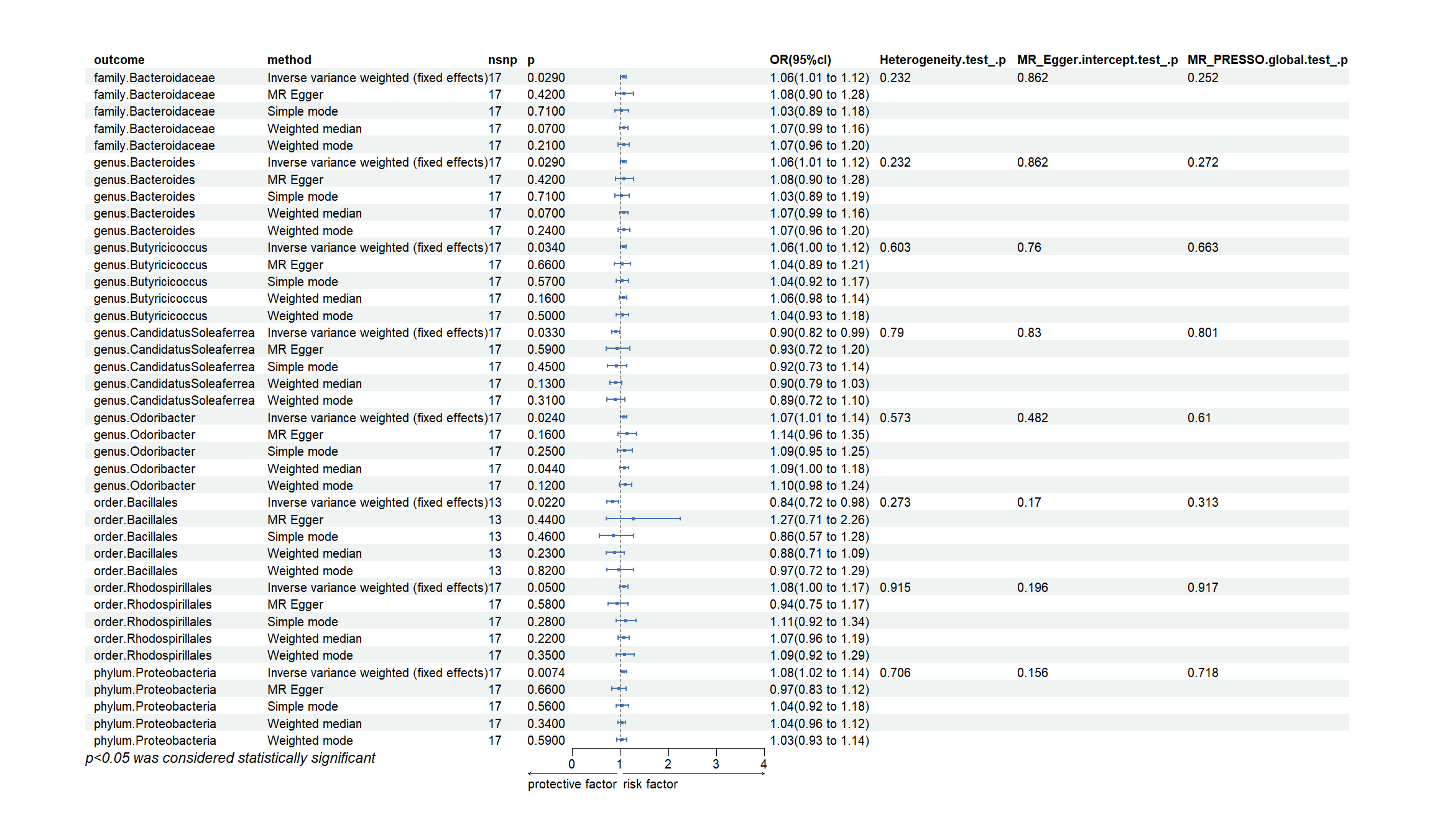

Supplement: Supplementary file 1 [file Data_Sheet_1.ZIP › Supplementary Material/Additional file 2/Supplementary Figure S3.tiff]
